# Evaluation of simple methods for regional mortality forecasts

**DOI:** 10.1186/s41118-018-0040-z

**Published:** 2018-09-27

**Authors:** Tom Wilson

**Affiliations:** Northern Institute, Charles Darwin University, Darwin, NT 0909 Australia

**Keywords:** Mortality forecast, Evaluation, Forecast error, Smoothness Index, Regions, Australia

## Abstract

**Background:**

In recent decades, considerable research effort has been dedicated to improving mortality forecasting methods. While making valuable contributions to the literature, the bulk of this research has focused on national populations—yet much planning and service delivery occurs at regional and local scales. More attention needs to be paid to subnational mortality forecasting methods.

**Objective:**

The objective of this study was to evaluate eight fairly simple methods of regional mortality forecasting, focusing specifically on the requirements of practising demographers in government and business.

**Data and methods:**

Data were sourced primarily from the Australian Bureau of Statistics. Retrospective mortality rate forecasts were produced for 88 regions of Australia for 2006–2016. Regional mortality forecast methods were evaluated on the basis of (i) input data requirements, (ii) ease of calculation, (iii) ease of assumption setting and scenario creation, (iv) plausibility of forecast death rates, (v) smoothness of forecast mortality age profiles, and (vi) forecast accuracy.

**Results:**

Two of the methods produced noticeably higher forecast errors than the others (National Death Rates and SMR Scaling). Five of the methods were judged to be similar in their overall suitability. Two were particularly easy to implement (Broad Age SMR Scaling and Broad Age Rate Ratio Scaling) and provide a good return on the data and effort required. Two others (Brass Relational and Mortality Surface) produced very smooth mortality age profiles and highly plausible death rates, though were relatively more complex to implement.

**Conclusion:**

The choice of mortality forecasting method is important for the accuracy of regional population forecasts. But considerations additional to accuracy are important, including those relating to the plausibility of the forecasts and the ease of implementation.

## Introduction

The improvement of mortality forecasting methods has been the focus of much research in demography and actuarial science in recent decades (Booth and Tickle 2008). There are now many sophisticated and complex mortality models to choose from which have been shown to generate quite accurate forecasts. Methods developed in recent years include various extensions of the well-known Lee-Carter model (e.g. Shang et al. 2011), functional data approaches (e.g. Hyndman and Ullah 2007), improved versions of long-established extrapolative methods (e.g. Ediev 2008), and combinations of multiple models (e.g. Kontis et al. 2017). A number of studies have compared the empirical characteristics and/or accuracy of selected recently developed mortality models (e.g. Booth et al. 2006, Cairns et al. 2009, Scherbov and Ediev 2016, Shair et al. 2017, Stoeldraijer et al. 2013, and Terblanche 2016).

These papers, and many others like them, all make important contributions to improving mortality forecasting. More accurate mortality forecasts mean more accurate population forecasts, especially at the older age groups where mortality is the dominant demographic process. And in many countries, populations in these age groups are growing rapidly (UN 2017a), as a result of larger cohorts flowing through to the older ages and declines in older adult mortality. The growth of the elderly population has important implications for governments, business, society, families, and the very old themselves (Australian Government 2015; Beard et al. 2012; Terblanche 2015; UN 2017b). Recent advances in mortality forecasting methods hopefully mean that today’s mortality forecasts are the most reliable ever (‘hopefully’ because we cannot be absolutely sure until forecasts are compared with actual outcomes many years into the future). Yet while the bulk of research on mortality forecasting focuses on national populations, much planning and service delivery occurs at regional and local scales. For example, the likely future growth of the elderly population in local communities informs planning for public hospitals, residential aged care, community care, and other goods and services designed for those at advanced ages.

This paper is concerned with subnational mortality forecasting, primarily at the scale of medium-sized subnational regions with populations mostly within the range 100,000 to 500,000. Population forecasts are commonly produced for this size of region in Australia. Relatively little attention in the mortality forecasting literature has been devoted to subnational mortality, and most of the models specifically designed for multiple areas (e.g. Bennett et al. 2015, Hyndman et al. 2013, and Li and Lee 2005) are best suited to large subnational populations such as states or provinces which have the requisite lengthy time series of input data. Many mortality forecasting methods developed in recent years are not easily transferable to smaller regions. The challenges involved in creating mortality forecasts at this scale include:

- The lack of long mortality rate time series (at least on a consistent set of geographical boundaries) required by many of the new mortality models;
- The small numbers of deaths and the resulting noisy (and in some cases, unrecognisable) mortality age profiles of smaller areas;
- The need to maintain plausible and consistent (or coherent) mortality relationships between areas (and sub-populations more generally);
- The complexity of many models. Although code is sometimes freely available, the statistical and programming knowledge required to understand and implement it can be substantial;
- The costs. The use of complex models will often require expensive data purchases and plenty of staff time in data adjustment, model fitting, validation, and so on.
- Other demands on forecasters’ time and skills. At the sub-state scale, migration is usually the dominant—and most uncertain—demographic process, so a considerable amount of time must be devoted to migration data preparation and assumption setting.

Nonetheless, in recent years, greater attention has been paid to the challenges of subnational mortality estimation and forecasting, often employing more sophisticated methods than in the past. For example, Alexander et al. (2017) used Bayesian methods to create robust local area mortality age profiles for US counties by sharing mortality information across space and time; Gonzaga and Schmertmann (2016) estimated local area mortality rate age schedules in Brazil with a combination of regression and the TOPALS relational model. Cairns et al. (2011) introduced a Bayesian age-period-cohort forecasting model which links a large population with a small sub-population; and Bennett et al. (2015) created several Bayesian spatio-temporal models to forecast the mortality of districts in England and Wales. This paper aims to contribute to this growing literature, but focusing primarily on the requirements of practising demographers in government and business. The objective of the paper is to evaluate eight methods of regional mortality forecast that do not require long time series of input data and can be implemented fairly easily (and in a spreadsheet). The methods were evaluated on the basis of six criteria of particular relevance to practising demographers: (i) input data requirements—more data means more time and expense in obtaining and handling it; (ii) ease of calculation—it is important that the time and expertise required to create the forecasts is not prohibitive; (iii) ease of assumption setting and scenario creation; (iv) plausibility of the forecast age-specific death rates—the age profiles should look believable and exclude implausible or impossible rates; (v) the smoothness of mortality age profiles—ideally they should represent the smooth underlying age pattern of mortality and be free of noise; and (vi) forecast accuracy—because ultimately, the accuracy of forecasts is important to users.

Mortality rate forecasts were produced for the 88 SA4 regions of Australia, medium-sized subnational regions in the official Australian Statistical Geography Standard (ABS 2011) which generally have populations within the range 100,000–500,000. They are the geographical areas for which population projections are commonly prepared in Australia by demographers in State Government and the private sector (e.g. QGSO (Queensland Government Statistician’s Office) 2016).

Following this introduction, the paper continues in the next section by briefly outlining several broad approaches to regional mortality forecasting and mentions examples of methods within each approach. In the section on ‘Data and Methods’, the forecasting models, input data, and evaluation criteria are described. The results of the evaluation are presented in the next section while the following section discusses the results, makes recommendations, and includes some concluding remarks.

## Approaches to regional mortality forecasting

Several broad approaches to the preparation of regional mortality forecasts can be distinguished, and these are summarised in Table 1. The first approach is just to assume all subnational regions experience the same projected mortality as the country as a whole or the state/province in which the region is located. This is the approach taken by a number of forecasters. For example, the regional population projections produced by the Irish Central Statistics Office (CSO (Central Statistics Office) (2013) take this approach on the grounds of small numbers of deaths in some regions and the minor impact of using region-specific mortality assumptions. Where regional mortality differences are trivial, this approach can be justified. However, the greater the regional variations in mortality, the more approximate this approach becomes, especially in the elderly age groups where mortality has a major impact on cohort size (Pittenger 1976).

**Table 1:** Some broad approaches to regional mortality forecasting

Approach	Examples
1. Use national or State, or region-type, mortality forecasts	Simply use national or State mortality forecasts for all regions (e.g. Pittenger 1976; CSO 2013), or apply forecasts for broad region types to all regions of that type
2. Trend regional mortality age schedules towards a long-run target	Trend regional mortality age profiles from the base period converging towards (or diverging away from) a very long-run set of target mortality rates (e.g. Van Hoorn and Broekman 1999 and Lanzieri 2016)
3. Apply models developed for single national populations to every region	For every region: • Fit a parameterised model schedule and extrapolate parameters • Use functional data models (e.g. Hyndman and Ullah 2007) • Fit new versions of extrapolative models (e.g. Ediev 2008) • Apply the Lee-Carter model (Lee and Carter 1992) or one its many extensions (e.g. Li et al. 2013)
4. Use multi-population models	• Lee-Carter extension for multiple populations (e.g. Li and Lee 2005) • Two-population age-period-cohort model (Cairns et al. 2011) • Product ratio method of Hyndman et al. (2013) • Bayesian approaches (e.g. Gongaza and Schmertmann 2016) • Multilinear component approach of Bergeron-Boucher et al. (2018)
5. Use a national or State mortality forecasts and create regional mortality forecasts via simple relationships	• Brass-type relational models (e.g. Brass 1971, Ewbank et al. 1983, and Murray et al. 2003) • National or State mortality age schedules scaled up or down according to the SMR (e.g. Giannakouris 2010 and NRS 2016) • TOPALS relational method using smoothed age profiles of rate ratios (de Beer 2012) • Assume regional death rates decline by the same proportional amount as forecast national death rates (Smith et al. 2013) • Mortality surface method (e.g. Wilson 2014, 2017) • National or State mortality age schedules scaled up or down according to regional/national mortality rate ratios (e.g. ONS 2016)

A related approach is to take account of regional mortality differences, but to trend base period regional mortality rates so that they gradually converge towards long-run target mortality rates. Or alternatively, they can be trended to diverge from their base period values either side of a target set of mortality rates. This ‘target and interpolation’ approach was taken by van Hoorn and Broekman (1999) in preparing regional European population projections as part of uniformity (convergence) and divergence scenarios. A similar approach of trending to a target is also currently applied by Eurostat in preparing national population projections for European Union member states. Mortality is projected by assuming partial convergence towards a set of very low rates created from long-term projections of the mortality of a group of lowest mortality EU nations (Lanzieri 2016, 2017).

A third approach is to just apply methods developed for individual national populations to all subnational regions. There are a large number of possible methods to choose from as covered in the reviews by Booth and Tickle (2008), Shang et al. (2011), Tabeau (2001), Terblanche (2016), and Wong-Fupuy and Haberman (2004), amongst others. But difficulties can arise in attempting to apply such methods to subnational regions, including a lack of sufficiently long time series of past data for model fitting and ‘noisy’ data patterns due to small numbers of deaths. In cases where the models *can* be successfully applied, then it is quite possible to obtain inconsistent and implausible forecasts across regions, including unlikely divergence, convergence, and trend crossovers over the forecast horizon.

A fourth broad approach is to use one of the sophisticated multi-population mortality forecasting models which has been created in recent years. These models were developed specifically to ensure consistency of forecasts between sub-populations, including between males and females, between countries in a multi-country forecast, and between states or provinces within a country. For example, Lee and Li (2005) extended the Lee-Carter model to handle multiple populations, presenting projection examples for the male and female populations of Sweden, and 15 low-mortality countries. This model has been used for province-level mortality forecasts by Statistics Canada (2010). Cairns et al. (2011) devised a two-population age-period-cohort model which links the mortality forecast of a large population with that of a small sub-population. Hyndman et al. (2013) developed the product-ratio functional forecasting method and illustrated its use with projections for Australian states and territories, and the male and female populations of Sweden. More recently, Bergeron-Boucher et al. (2018) employed multilinear component techniques to create consistent mortality projections for Canada’s provinces and territories. Other multiple population contributions include those by Bergeron-Boucher et al. (2017), Enchev et al. (2017), and Gonzaga and Schmertmann (2016). All of these models ensure mortality forecasts maintain sensible and plausible relationships between subpopulations.

A fifth general approach is to take national, or state or province-level, mortality forecasts and create regional forecasts from them via simple relationships. Generally, these relationships are assumed to be time-invariant, an assumption which is generally reasonable in the short to medium term (Kibele et al. 2015; Oosse 2003). This broad approach is taken by a number of statistical agencies. One simple method is to calculate regional base period age-sex-specific death rates and apply the proportional change in rates over time from the national mortality forecast (Smith et al. 2013). Another option is to scale forecasts of national mortality rates using region-specific Standardised Mortality Ratios (SMRs) calculated for a recent period. The data requirements are low and the calculations simple. This method was employed in the 2008-based regional population projections for the European Union (Giannakouris 2010). Projections for local areas of Scotland are also prepared using this method except that separate SMRs are calculated by sex and three broad age groups (0–59, 60–79, and 80+) (NRS (National Records of Scotland) 2016). Alternatively, ratios of regional age-specific death rates to national age-specific death rates can be calculated, and then multiplied by national mortality forecasts in the projection computations. This rate ratio approach is simple and easy to apply, and a version of it is used by the UK Office for National Statistics for projecting mortality in subnational areas of England (ONS (Office for National Statistics) 2016). ONS applies regional/national age-specific mortality rate ratios averaged over the most recent 5 years, updating them as the forecasts progress. There is some capping of ratios to avoid extreme values. A refined version of the rate ratio approach is de Beer’s TOPALS (TOol for Projecting Age-specific rates using Linear Splines) method which involves smoothing rate ratio age profiles using linear splines (de Beer, 2012).

Slightly more complex relational models include the Brass relational model (Brass, 1971) and its extensions (e.g. Ewbank et al. 1983 and Murray et al. 2003). The Brass model relates life table *l*_*x*_ values from a national (or model) population to those for regions (or other subpopulations). Two parameters describe the relationship of regional mortality to national mortality, and they are used to forecast regional mortality assuming the relationship remains constant. An alternative method is to obtain region-specific mortality rates from a national mortality surface consisting of past and projected life tables which span a wide range of mortality conditions. Effectively, it acts as a set of model life tables. Regional mortality is projected in terms of life expectancy at birth, and then death rates which correspond to the assumed life expectancy are selected from the mortality surface (Wilson 2014, 2015). This type of method is useful when forecasters wish to formulate regional mortality assumptions in terms of the ‘headline’ indicator of life expectancy at birth.

Other approaches, such as having base period regional mortality rates remain constant into the future, are generally not plausible assumptions and are not considered in this paper. Purely judgemental approaches are also not considered.

## Data and methods

### Data

National deaths and population estimates for 1921 to 2012 by single years of age were obtained from the Human Mortality Database (HMD (Human Mortality Database) 2017) with more recent deaths and population estimates taken from Wilson and Terblanche (2018). Both datasets are derived from original Australian Bureau of Statistics data. These data were used to calculate national (Australian) life tables. Regional deaths and Estimated Resident Populations by sex and 5-year age group for the periods 2001–2006, 2006–2011, and 2011–2016 were obtained from the Australian Bureau of Statistics. These data enabled the calculation of age-sex-specific death rates and abridged sex-specific life tables for each of the SA4 regions. Regional life expectancy at birth in 2001–2006 ranged from 75.1 to 85.5 years for females and 68.7 to 81.9 years for males. Period-cohort occurrence/exposure death rates, used in the forecast calculations, were calculated from life table _*n*_*L*_*x*_ values as

$$ {d}_{pc}=\left({{}_5L}_x-{{}_5L}_{x+5}\right)/\frac{5}{2}\left({{}_5L}_x+{{}_5L}_{x+5}\right). $$

### Forecasting models

Eight fairly simple subnational mortality forecasting models implemented by statistical agencies or researchers in recent years were chosen for evaluation. All fall within approaches 1 and 5 listed in Table 1 and are linked to a national mortality forecast in some manner. The reason for choosing models within these broad approaches only is because of the practical emphasis of the paper: the selected models are relatively simple in that they do not require lengthy time series of input data and are not too difficult to implement in a spreadsheet. All models were estimated over a base period of 2001–2006 and used to create mortality rate ‘forecasts’ by sex and 5-year period cohorts for the periods 2006–2011 and 2011–2016. The year labels refer to periods from 1 July of 1 year to the 30 June 5 years later. Period cohorts are parallelogram age-time spaces on the Lexis diagram which show the ages of cohorts as they age over time (Rees and Woods 1986 p. 306), e.g. the period cohort aged 20–24 in 2001 which ages to 25–29 by 2006 (written as ‘20–24 to 25–29’).

All regional mortality forecast models made use of a 2006-based national life table forecast by sex and single years of age. This was produced using Ediev’s (2008) method which applies linear extrapolation to the logarithms of age-specific death rates, subject to consistency and plausibility constraints. The fitting period varies by age-sex group and is determined automatically as the period which provides the best fit to the mortality rate trend for that age-sex group. Terblanche (2016) showed this model to be about as accurate for Australia as the Booth-Maindonald-Smith version of the Lee-Carter model (Booth et al. 2002). For the purposes of this evaluation, minor adjustments to ensure regional death rates were consistent with national rates were not made because this constraint is often applied to forecast death numbers during the course of forecast calculations.

The eight regional models are as follows.^1^ The first, *National Death Rates*, is the simplest approach and just assumes that every subnational area’s mortality rates can be approximated by national (Australian) rates. Thus:

$$ {d}_{pc,s}^i(y)={d}_{pc,s}^{nat}(y) $$

where *d* refers to death rate, *pc* period-cohort, *s* sex, *y* projection interval, *i* subnational area, and *nat* the national population. There are no regional input data requirements.

The second model, *SMR Scaling*, multiplies forecast national death rates by a subnational Standardised Mortality Ratio (SMR) (Giannakouris 2010). Thus:

$$ {d}_{pc,s}^i(y)={d}_{pc,s}^{nat}(y)\kern0.5em {SMR}^i $$

An SMR is the ratio of the observed number of deaths in a region to the ‘expected’ number if the age-sex-specific death rates from a national (or standard) population were to apply. The regional input data therefore typically consists of base period total deaths and age-sex-specific populations-at-risk. In fact, for Australia, less data are required if SA4 regions are used because the Australian Bureau of Statistics publishes Indirectly Standardised Death Rates (ISDRs) for these areas (ABS 2016). SMRs can be calculated as ISDRs divided by the national Crude Death Rate. For this study, it was assumed that SMRs for the base period (the last 5 years) remained unchanged over the forecast horizon.

A more refined version involves SMR scaling by broad period cohort or age group ranges (NRS 2016). This third model is referred to as *Broad Age SMR Scaling* and can be written as:

$$ {d}_{pc,s}^i(y)={d}_{pc,s}^{nat}(y)\kern0.5em {SMR}_{PC,s}^i $$

where *PC* (capitalised) denotes broad period cohorts. In this study, they were, for females, birth–74 to 0–79, 75–84 to 80–89, and 85+ to 90+; and for males, birth–64 to 0–69, 65–79 to 70–84, and 80+ to 85+. Again, SMRs were assumed to be time-invariant for the purposes of this evaluation. The regional input data required to calculate the SMRs are base period regional deaths by sex and broad age group (or period cohort), and age-sex-specific populations-at-risk.

The fourth model obtains regional death rates from a *Mortality Surface*^2^ created from a range of historical and forecast national life tables (Wilson 2015). The surface consists of life table _*n*_*L*_*x*_ values by sex and age over time, with the temporal range extending far enough so that it covers all possible future mortality levels for all subnational regions. In practice, it uses national life tables from several decades into the past to those for well over a century into the future. The mortality surface effectively acts as a series of model life tables (Moultrie and Timaeus 2013) for the subnational regions. It assumes that all regions follow a mortality trajectory described by the national mortality surface, but from different starting points and at their own pace.

Regional life expectancy at birth assumptions by sex is required to determine each region’s place in the mortality surface. They can be created by multiplying independent national life expectancy forecasts by a regional scaling factor (which is the base period regional to national life expectancy ratio), i.e.:

$$ {e}_{0,s}^i(y)={e}_{0,s}^{nat}(y)\ {sf}_s^i $$

where *e*_0_ is life expectancy at birth and *sf* is the *e*_0_ scaling factor. Base period regional life expectancy values were calculated using abridged life tables. The scaling factors were assumed to remain fixed at their base period values in this study. Then, _*n*_*L*_*x*_ values can be selected from the point on the mortality surface which corresponds to the regional life expectancy at birth assumption, i.e. where:

$$ {e}_{0,s}^i(y)=\frac{T_{0,s}^{MS}}{\mathrm{100,000}} $$

where *MS* denotes mortality surface and *T*_0_ the total number of _*n*_*L*_*x*_ person-years lived in the life table population above age 0. The _*n*_*L*_*x*_ values are then used to calculate period-cohort death rates. The regional input data required for this model are local and national life expectancy at birth estimates for the base period.

The fifth model is the *Brass relational* model which relates regional mortality to national mortality via two parameters (Brass 1971; Sloggett 2015). The model is based on a logit transformation of national and regional life table populations at exact ages, *l*_*x*_. It is calculated as:

$$ \mathrm{logit}\ {l}_x=\frac{1}{2}\ \ln \frac{1-{l}_x}{l_x}. $$

The logit transformation enables regional mortality to be modelled as a function of national mortality by linear regression. To simplify the expression, if:

$$ {Y}_x^i=\mathrm{logit}\ {l}_x^i $$

then regional mortality can be expressed as:

$$ {\widehat{Y}}_x^i={\alpha}^i+{\beta}^i\ {Y}_x^{nat}. $$

The *α* variable describes the overall level of regional mortality relative to the standard, while *β* describes how younger and older mortality vary relative to the standard. Both *α* and *β* values were assumed to remain constant into the future. The input data consist of national and regional sex-specific life tables for the base period.

The sixth model, *Rate Ratio Scaling*, multiplies projected national death rates by regional rate ratios (ONS (Office for National Statistics) 2016). These comprise ratios of regional to national death rates by sex and period cohort. Thus:

$$ {d}_{pc,s}^i(y)={d}_{pc,s}^{nat}(y)\kern0.5em r{r}_{pc,s}^i $$

where *rr* denotes rate ratio. Rate ratios were assumed to remain fixed at their base period values. The input data required to calculate rate ratios consist of regional and national sex and period-cohort death rates for the base period. This model is numerically equivalent to the approach in which regional base period death rates are multiplied by the ratio of national forecast mortality to base period national mortality (Smith et al. 2013).

The seventh model, *Broad Age Rate Ratio Scaling*, is a variation of the previous one, except that broad period-cohort ranges are used to effectively provide smoothing to the rate ratios (NRS (National Records of Scotland) 2016). It can be written as:

$$ {d}_{pc,s}^i(y)={d}_{pc,s}^{nat}(y)\kern0.5em r{r}_{PC,s}^i $$

where *PC* refers to the broad period-cohort ranges of, for females, birth–74 to 0–79, 75–84 to 80–89, and 85+ to 90+; and for males, birth–64 to 0–69, 65–79 to 70–84, and 80+ to 85+. The input data consist of national and regional broad age group (or period-cohort) death rates.

The eighth model is a refinement of Rate Ratio Scaling. It is a simplified version of de Beer’s (2012) *TOPALS* (TOol for Projecting Age-specific rates using Linear Splines) approach which provides a simple but effective way of smoothing mortality age profiles. In the application here, projected national death rates are multiplied by regional rate ratios which have been smoothed using linear splines. Thus:

$$ {d}_{pc,s}^i(y)={d}_{pc,s}^{nat}(y)\kern0.5em {\overset{\sim }{rr}}_{pc,s}^i $$

where $\overset{\sim }{rr}$ denotes smoothed rate ratios. In this study, the linear spline knots were selected at period-cohorts birth to 0–4, 10–14 to 15–19, 25–29 to 30–34, 40–44 to 45–49, 55–59 to 60–64, 70–74 to 75–59, and 85+ to 90+ with the other rate ratios calculated by linear interpolation. The smoothed rate ratios were assumed to remain constant over time (an assumption which differs from de Beer’s (2012) use of projected rate ratios). The input data are the same as for the Rate Ratio Scaling model.

### Criteria for assessment

#### (i) Input data requirements

Input data requirements for each of the various mortality forecasting methods were assessed by compiling a table of both national-level and regional-level data inputs. At the regional scale the number of input data cells per subnational region was noted.

#### (ii) Ease of calculation

Ease of calculation is difficult to assess because it varies according to each forecaster’s skill set and available software, staff, and other resources. A personal qualitative judgement of ‘easy’, ‘moderate’, or ‘complex’ was made taking into account the numbers of cells per region in the Excel workbook used by this author to complete the calculations, whether any programming was required, and the conceptual complexity of the calculations. Different researchers may use alterative calculations steps and take different views of the complexity of the methods, of course. Nonetheless, this basic three-category assessment provides an approximate guide to the relative ease of calculation of the various methods.

#### (iii) Ease of assumption setting

Ease of assumption setting and scenario creation is also challenging to assess quantitatively and was also assessed by judgement. Assumption setting was considered to be ‘easy’, ‘moderate’, or ‘difficult’. Some models allow more flexibility in their assumptions while others effectively embed fixed assumptions and relationships within them. Those models which would require extensions or modifications to include region-specific changes to assumptions were classified as ‘difficult’.

#### (iv) Plausibility

A very simple plausibility test of the forecast regional mortality age profiles was applied. It was simply a count of the number of regions with mortality rate age profiles in which an age-specific death rate fell outside the acceptable range of greater than zero and up to 0.4. Rates greater than 0.4 in a 5-year interval cohort-component projection model based on the linear integration hypothesis will result in more deaths than in the initial population (Hoem and Funck Jensen 1982). Rates of exactly zero are unlikely to represent the true underlying death rate at any age.

#### (v) Age profile smoothness assessment

In general terms, the rate at any particular age in a smooth age profile of underlying demographic rates is generally closely related to those at ages either side of it. In a smooth age profile of mortality, the rate at age *a* is close to the exponential of the mean of the natural log rates at ages *a* − 1 and *a* + 1, at least for most of the adult ages. The difference between the observed rate at age *a* and the ‘expected’ smooth rate can be calculated as:

$$ {diff}_a^i={\mathrm{rate}}_a^i-{\mathrm{smooth}}_a^i $$

and in the case of mortality:

$$ {diff}_a^i={\mathrm{rate}}_a^i-\exp \left[\frac{1}{2}\ \left(\ln \left({\mathrm{rate}}_{a-1}^i\right)+\ln \left({\mathrm{rate}}_{a+1}^i\right)\right)\right]. $$

The values of *diff* can be calculated for all age groups except the first and last. If a region’s age profile of rates is quite jagged the *diff* values will be quite large. The sum of absolute *diff* over all ages is then related to the total value of rates over all ages *a* (except the first and last age groups) and both sexes to give an overall measure of jaggedness. A Jaggedness Index may be calculated:

$$ {J}^i=\frac{\sum \limits_a\left|{diff}_a^i\right|}{\sum \limits_a{\mathrm{rate}}_a^i}\ 100. $$

The higher the Jaggedness Index, the more jagged (or noisy) the age profile of rates.

A more refined jaggedness measure could be created which measures the extent to which any region’s age profile of rates deviates from a smooth model age schedule of rates (Peleg and McClements 1997) through measures such as the sum of squared residuals or root mean square error. But that would require specifying and fitting a model. The measure described here, while approximate, is simple and easy to calculate and does not impose any predetermined mortality age profile.

Alternatively, where the emphasis is on smoothness, as it is here, a Smoothness Index may be preferred. It can be calculated simply as 100 minus the Jaggedness Index:

$$ {S}^i=100-{J}^i $$

where a value of 100 represents a perfectly smooth age profile. The Smoothness Index is reported for the regional mortality age profiles projected in this study.

#### (vi) Forecast error assessment

Error (*E*) is defined as the forecast death rate minus the observed death rate. Positive errors indicate death rates which were over-forecast; negative errors mean the death rates were under-forecast. Two main error measures are used in this paper. First, to assess how well the level of mortality was forecast for individual regions overall, the signed errors for death rates by sex and period cohort were summed over sex and period cohort. The signed errors will usually partly cancel out, giving a measure of the overall magnitude of error in forecasting mortality across all ages and both sexes. Taking the absolute value of this gives the regional Absolute Total Error (ATE), i.e.

$$ {\mathrm{ATE}}^i=\left|{\sum}_s{\sum}_{pc}{E}_{pc,s}^i\right|. $$

Second, to assess how close forecast mortality rate *age profiles* for individual regions came to the observed rates, the absolute error for each death rate by sex and period cohort was summed over all sex and period-cohort groups in that region. This gives the regional Total Absolute Error (TAE), i.e.

$$ {\mathrm{TAE}}^i={\sum}_s{\sum}_{pc}\left|{E}_{\mathrm{p}c,s}^i\right|. $$

Percentage errors have not been calculated. For the evaluation of death rates, which vary enormously in value by age, the error of the death rate is more useful. Higher death rates at the oldest ages will likely suffer from larger errors and will therefore contribute more to errors in forecasting numbers of deaths. This is preferable to the equal weighting of error when percentage errors are summed: a 10% error at the highest ages is far more of a problem in population forecasting than a 10% error in the early teenage years where death rates are very low.

## Evaluation

### Input data requirements

Input data requirements for the eight mortality models are summarised in Table 2. The number of input data cells used in this study is summarised in the table in terms of *r*, where *r* denotes the number of subnational regions. All methods require national mortality forecasts and most also need base period mortality data to calculate various regional/national ratios or similar parameters. Importantly, none of the methods require lengthy time series of regional mortality rates. The lowest input data requirements are obviously for the National Death Rates approach in which all regions are assumed to experience national death rates throughout the projection horizon. For the other methods, varying amounts of regional data are required, though the exact amount depends on the detail of published regional mortality statistics and the exact implementation of each method.

**Table 2:** Input data requirements of the eight regional mortality forecasting methods

Method	National input data	Regional input data
National Death Rates	National forecast death rates	None (Data cells = 0)
SMR Scaling	National base period and forecast death rates	Regional base period SMRs: total deaths by region; ERPs^(1)^ by age and sex by region (Data cells = 41*r*) ^(2)^
Broad Age SMR Scaling	National base period and forecast death rates	Regional base period SMRs: deaths by sex and broad age group by region; ERPs by sex and age group by region (Data cells = 46*r*)
Mortality Surface	National mortality surface of past and forecast _*n*_*L*_*x*_ values	Projected *e*_0_ by sex by region.^(3)^ (Data cells = 2*r*)
Brass Relational	National base period and forecast *l*_*x*_ values	Regional base period *l*_*x*_ values: deaths and ERPs by age and sex to create life tables. (Data cells = 80*r*)
Rate Ratio Scaling	National base period and forecast death rates	Regional base period rate ratios: deaths and ERPs by sex and age group by region (Data cells = 80*r*)
Broad Age Rate Ratio Scaling	National base period and forecast death rates	Regional base period broad age rate ratios: deaths and ERPs by sex and broad age group by region (Data cells = 12*r*)
TOPALS	National base period and forecast death rates	Regional base period rate ratios: deaths and ERPs by sex and age group by region. (Data cells = 80*r*)

Notes: (1) ERP = Estimated Resident Population. (2) *r* = number of regions. (3) If base period regional *e*_0_ values are not available they will need to be calculated, requiring deaths and ERPs by age and sex to create life tables. Regional *e*_0_ forecasts can be created by assuming they remain a constant proportion of an independent national *e*_0_ forecast throughout the forecast horizon

For the SMR Scaling method, base period SMRs must be calculated. This requires *r* + 40*r* regional input data cells consisting of r total death counts for the base period to form the numerator of the SMR, and 40*r* age-sex groups of regional ERPs used to calculated the ‘expected’ deaths if national death rates applied. In the Broad Age SMR Scaling method, SMRs were calculated for three broad age groups by sex, requiring deaths for six broad age-sex groups and 40*r* age-sex groups of regional ERPs used to calculate the ‘expected’ deaths.

In the Mortality Surface approach, most of the input data and data preparation occurs at the national scale in creating the national mortality surface itself. Relatively little *regional* input data is required. Just base period regional life expectancy at birth by sex is needed (2*r* data cells), though if regional life expectancies are unavailable they will have to be calculated from age-sex-specific death rates and the input data requirements will be higher. Regional life expectancy forecasts are calculated by multiplying base period life expectancy regional/national scaling factors by independent national life expectancy forecasts.

In the Brass Relational model, regional *l*_*x*_ values by age and sex are required for the base period in order to calculate the alpha and beta parameters which relate regional mortality age profiles to the national profile. Regional input data to calculate the necessary life tables consist of deaths by age group and sex (40*r*) and ERPs by age group and sex (40*r*).

The Rate Ratio Scaling method requires the calculation of base period regional/national ratios of death rates. The input data consists of regional deaths by age and sex (40*r*) and ERPs by age and sex (40*r*). In the Broad Age Rate Ratio Scaling method, rate ratios were calculated for three broad age groups by sex, requiring deaths for 6*r* broad age-sex groups and 6*r* age-sex groups of regional ERPs. Similarly, for the TOPALS approach, the input data consists of regional deaths by age and sex (40*r*) and ERPs by age and sex (40*r*) to create rate ratios.

### Ease of calculation

All mortality forecasts can be prepared in an Excel workbook (though some methods are best operationalised using some VBA coding). Table 3 summarises this author’s evaluation of each method’s relative ease of calculation. The National Death Rates approach is the easiest because no regional projection calculations are required. National mortality forecasts can be calculated from the many programmes freely available on the web, or they can be obtained from the relevant national statistical office. The SMR Scaling and Broad Age SMR Scaling methods are similarly classified as easy because the calculations are basic and can be completed quickly.

**Table 3:** Relative ease of calculation of the eight regional mortality forecast methods

Method	Ease of calculation
National Death Rates	Easy (no regional calculations needed)
SMR Scaling	Easy
Broad Age SMR Scaling	Easy
Mortality Surface	Complex
Brass Relational	Complex
Rate Ratio Scaling	Easy
Broad Age Rate Ratio Scaling	Easy
TOPALS	Moderate

Source: author’s assessment

The Rate Ratio and Broad Age Rate Ratio methods are also easy to calculate. The calculations just involve dividing regional death rates by national death rates to obtain rate ratios. The rate ratios are multiplied by forecasts of national death rates to yield regional projected death rates.

The TOPALS method is classified as moderate because, in additional to rate ratio calculations, it requires linear interpolation, and some judgement about the most appropriate age groups to form the linear spline knots and whether the rate ratio values for those knots require smoothing. But overall, it is not too difficult a method to implement.

The Mortality Surface approach is relatively complex and involves a number of steps. In this study, it was implemented with a VBA subroutine, though it is possible to set out the calculations in a spreadsheet in a number of steps without any code. First, the national mortality surface was created using past and projected life table _*n*_*L*_*x*_ values. Then, base period scaling factors of regional to national life expectancy at birth values by sex were calculated. Regional life expectancy forecasts were created by multiplying national life expectancy forecasts by the scaling factors. Appropriate _*n*_*L*_*x*_ values were then picked out of the mortality surface corresponding to each projected life expectancy at birth value. This is the most complex part because Excel formulas must be created to select _*n*_*L*_*x*_ values which correspond with each projected life expectancy at birth assumption. Period-cohort death rates can then be obtained from the interpolated _*n*_*L*_*x*_ values.

The Brass Relational method is also relatively complex. It requires base period *l*_*x*_ values for both national and regional populations. The logits of regional *l*_*x*_ values are regressed against those of the national population to obtain the alpha and beta parameters using Excel = intercept() and = slope() commands. These parameters are assumed fixed into the future and are used to obtain logits of regional *l*_*x*_ values in the future. These are then converted back to *l*_*x*_, and then using life table calculations _*n*_*L*_*x*_ values can be obtained. Finally, period-cohort death rates are obtained from the _*n*_*L*_*x*_ values.

The ease-of-calculation evaluation presented here is a subjective assessment, and it is of course the case that such an assessment will vary from one analyst to another. It will also depend to a large extent on whether the calculations are undertaken from scratch, or whether a pre-prepared spreadsheet template, script, or programme is available to perform the calculations.

### Ease of assumption setting and scenario creation

Table 4 summarises the ease of assumption setting and scenario creation for the various methods. The National Death Rates method, by definition, sets all regional death rates equal to the national death rate forecasts, so it is not really possible to incorporate alternative regional assumptions or scenarios. The SMR Scaling, Broad Age SMR Scaling, Brass Relational and Mortality Surface methods can incorporate region-specific assumptions through alternative SMRs, life expectancy at birth inputs, and *α* and *β* parameters (Brass Relational model). The remaining methods have no obvious way of incorporating alternative assumptions or scenarios. None of the methods contain in-built mechanisms to easily create alternative assumptions or scenarios by adjusting, for example, a mortality convergence parameter. Additional scenario creation work would be necessary to achieve this (e.g. Rees et al. 2012).

**Table 4:** Relative ease of assumption setting and scenario creation of the eight regional mortality forecast methods

Method	Ease of assumption setting
National Death Rates	Difficult (not really possible)
SMR Scaling	Moderate
Broad Age SMR Scaling	Moderate
Mortality Surface	Moderate
Brass Relational	Moderate
Rate Ratio Scaling	Difficult
Broad Age Rate Ratio Scaling	Difficult
TOPALS	Difficult

Source: author’s assessment

### Plausibility of projected regional death rates

**Fig. 1:**
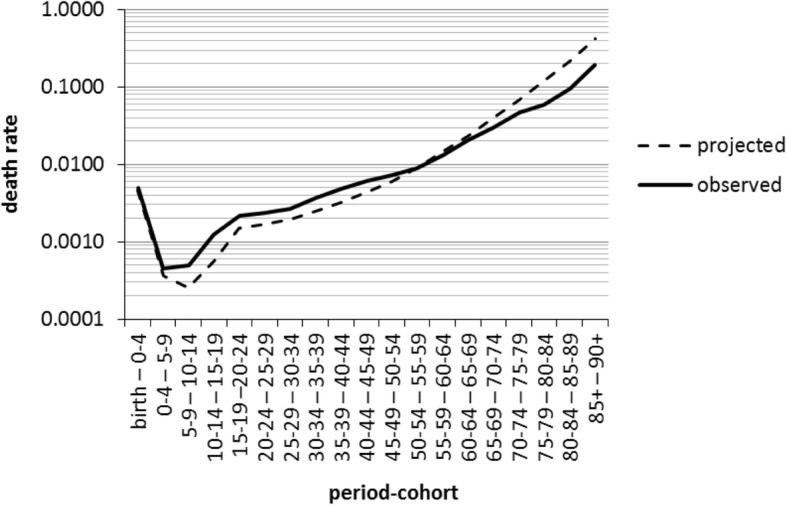
Observed and projected (SMR Scaling) mortality rates for Northern Territory Outback males, 2006–2011. Source: observed mortality age schedule calculated from ABS data

Only one method produced a forecast death rate which exceeded 0.4. The SMR Scaling method produced a death rate above 0.4 in the highest age period cohort for Northern Territory Outback males. This is a risk with the SMR Scaling method in regions with high SMRs because national mortality rates at every age are multiplied by the one SMR to give the estimated regional mortality rates. Figure 1 illustrates observed Northern Territory Outback male death rates in 2006–2011 together with those projected by the SMR Scaling method. In this case, scaling by a one-size-fits-all SMR results in death rates which are too low in the younger and middle age groups and too high in the older age groups. The Broad Age SMR Scaling method can, in theory, also produce death rates of greater than 0.4 but because SMRs are calculated for several broad age ranges this problem is much less likely to occur in practice.

No methods produced death rates of zero in this study, but it is worth noting that the Rate Ratio Scaling method is susceptible to producing them. When there are no deaths in one particular period-cohort in a region in the base period the rate ratio will be zero, and the resulting projected death rate will therefore also be zero. The theoretical underlying death rate is unlikely to be zero. Regions with smaller populations, with small numbers of deaths, will be at greater risk of this occurring. The Broad Age Rate Ratio Scaling is much less prone to this issue due to the use of broad age group rate ratios which are less likely to be zero. Similarly, the smoothing of rate ratios in the TOPALS method makes the possibility of a zero death rate less likely.

The National Death Rates approach, where all projected regional death rates are set equal to national death rates, is a very safe option in that all regional death rates will be plausible (so long as the national mortality forecast is competent). The Mortality Surface method is also safe in that all projected regional death rates will fall within the range of the national mortality surface. The Brass Relational method is a similarly good option in this regard because all regional mortality age profiles consist of adjusted versions of the national profile using level (α) and slope (β) parameters.

### Smoothness of projected mortality age profiles

**Fig. 2:**
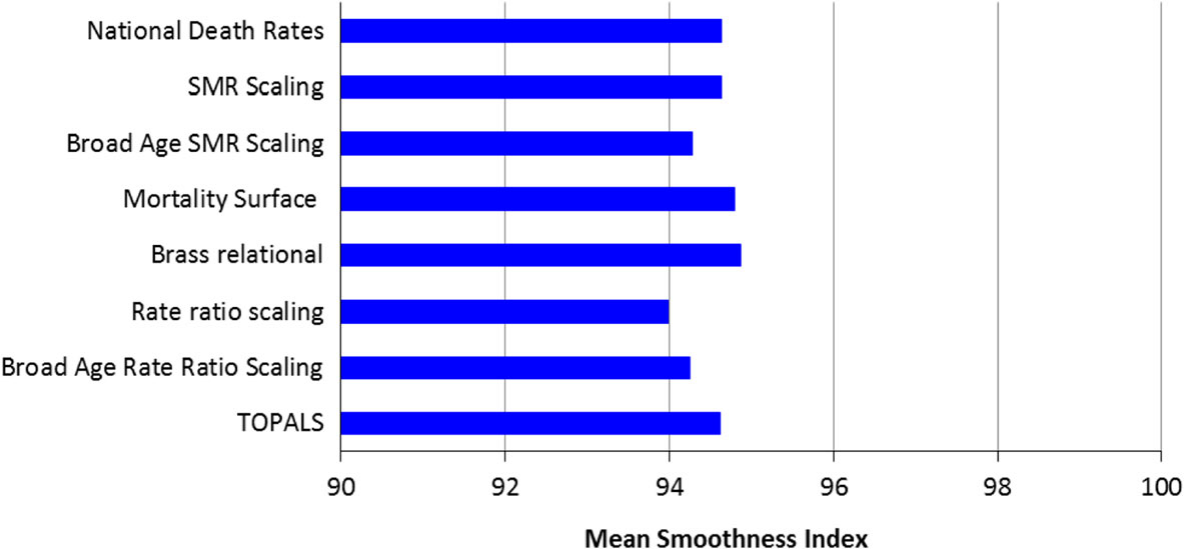
Mean smoothness of forecast mortality rate age profiles across the 2006–11 and 2011–2016 periods across all Australian regions. Note: Mean Smoothness Index values were calculated for each method by averaging the Smoothness Index across all regions and both forecast periods

The smoothness of the forecast mortality age profiles did not vary greatly between methods. Figure 2 shows the Smoothness Index for mortality age profiles averaged across all regions and both 2006–2011 and 2011–2016 periods. The Rate Ratio Scaling method produced slightly less smooth mortality age profiles than the other methods, a finding which is unsurprising given that rate ratios incorporate noise from the base period regional mortality age profiles.

The Brass Relational and Mortality Surface methods generated slightly smoother age profiles than the others. The Brass model introduces smoothing by using the national mortality age profile adjusted to the level and slope of the regional age profile through the alpha and beta parameters. The Mortality Surface method makes use of a national surface of smoothed age-specific death rates and extracts a set of age-specific death rates which match the life expectancy at birth assumption.

The four methods National Death Rates, SMR Scaling, Mortality Surface, and Brass Relational will always produce relatively smooth mortality age profiles because they are very closely or directly based on national mortality age profiles. The other four methods are likely to produce age profiles of varying smoothness depending on numbers of deaths (related to region population size) and how many broad age groups, or spline knots, are used (the fewer the number, the greater the smoothness).

### Forecast accuracy

Table 5 summarises the forecast accuracy of the eight models at 50% (median) and 95% of the error distribution for Absolute Total Error (overall mortality) and Total Absolute Error (mortality age profiles). Both error measures clearly show that National Death Rates yielded the most inaccurate mortality forecasts. SMR Scaling achieved a reasonable median Absolute Total Error but its 95% value indicates a longer tail of high errors than most other methods. Both National Death Rates and SMR Scaling were the least successful in forecasting mortality age profiles, as the right-hand columns of the table demonstrate (Total Absolute Error).

**Table 5:** Summary of death rate errors across all regions of Australia and both 2006–2011 and 2011–2016 periods

Method	Absolute Total Error (overall mortality)		Total Absolute Error (mortality age profiles)	
	Median	95%	Median	95%
National Death Rates	0.035	0.105	0.054	0.117
SMR Scaling	0.020	0.091	0.048	0.128
Broad Age SMR Scaling	0.016	0.066	0.040	0.087
Mortality Surface	0.017	0.067	0.038	0.083
Brass Relational	0.020	0.072	0.038	0.088
Rate Ratio Scaling	0.018	0.065	0.039	0.087
Broad Age Rate Ratio Scaling	0.021	0.074	0.042	0.092
TOPALS	0.019	0.071	0.040	0.085

Note: Absolute Total Error and Total Absolute Error measures were calculated for each method taking into account errors across all regions and both forecast periods

Amongst the remaining methods, none was clearly superior to any of the others. For overall mortality the best method, by a small margin, was Broad Age SMR Scaling, followed closely by several other methods. For mortality age profiles, the best, again by small margins, were the Mortality Surface and Brass Relational methods.

**Fig. 3:**
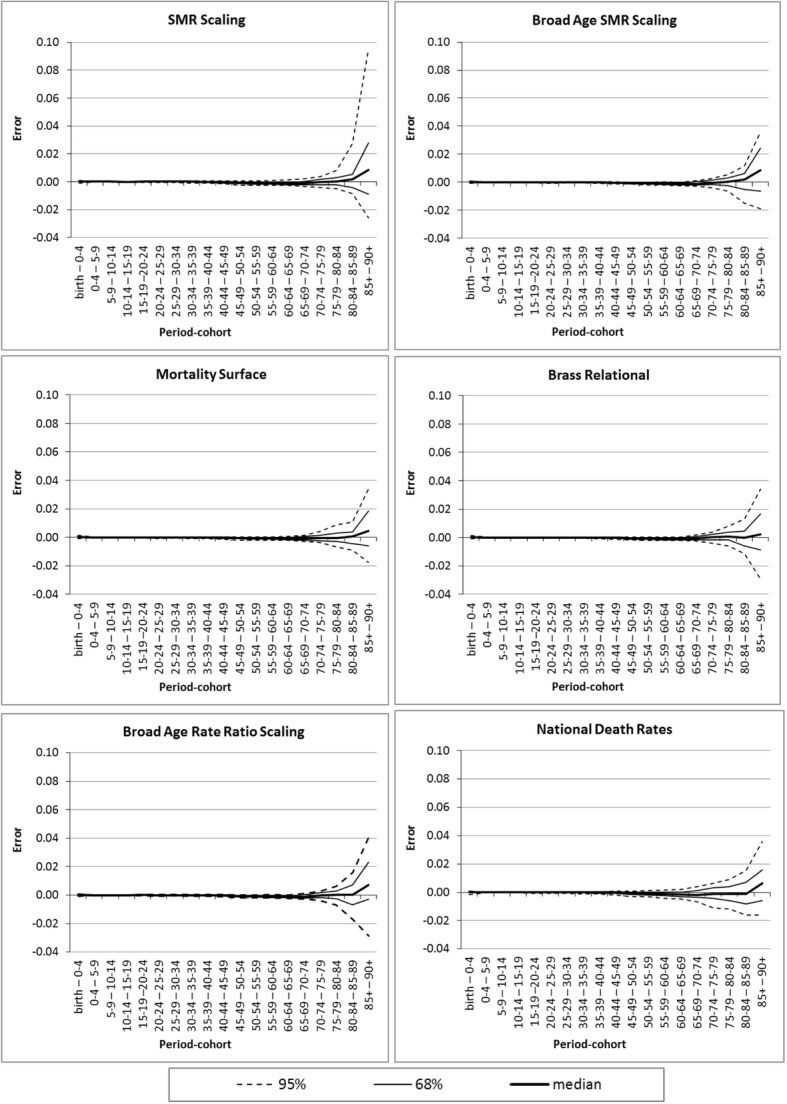
The distribution of mortality rate errors for males by period cohort in 2011–2016, across Australian regions, for selected regional mortality forecasting models

An illustration of the distributions of forecast errors by period cohort is shown in Fig. 3. For reasons of space the graphs show errors for males in 2011–2016 and for six methods only. Given that death rates increase rapidly with age, it is not surprising to see the largest errors at advanced ages. With SMR Scaling the range of errors at the highest ages was considerable, and skewed towards pessimistic over-forecasts of mortality. The simple disaggregation of the SMR to broad age-sex groups resulted in a much narrower error distribution (Broad Age SMR Scaling).

Error patterns for the Mortality Surface, Brass Relational, and Broad Age Rate Ratio Scaling models proved fairly similar (as were those for the Rate Ratio and TOPALS methods, not shown). Bias (whether forecasts were, overall, too high or too low) was lowest in the Brass Relational and Mortality Surface methods. The error distribution for National Death Rates proved similar to that of many others at the highest period-cohort, but the key difference is larger errors in the younger elderly ages (60s and 70s) than the other methods. This is how it received the high Total Absolute Error values shown in Table 5.

## Discussion and conclusion

This paper has reported on an assessment of eight simple methods for forecasting regional mortality rates, with an empirical assessment undertaken for regions of Australia. The methods were evaluated against six criteria: input data requirements, ease of calculation, ease of assumption setting and scenario creation, plausibility of forecasts, smoothness of mortality age profiles, and forecast accuracy. Given the findings presented above, which of the forecasting models work best for subnational regions and can be recommended? Table 6 presents a summary of the evaluation.

National Death Rates is an attractive approach in terms of input data, ease of calculation, plausibility and smoothness of migration age profiles. But in the evaluation it performed poorly in terms of accuracy. Unless regional variations in mortality are negligible it is best avoided. SMR Scaling also possesses attractive features, including low data requirements, simple calculations, and smooth age profiles. But it can generate 5-year age group death rates which exceed plausible limits, and its forecast accuracy proved to be disappointing. It is also not recommended. Rate Ratio Scaling is also simple to implement but is susceptible to producing death rates of zero where populations are small, and its age profiles are the least smooth of all methods studied. Other methods are probably better choices.

The remaining five methods all produced fairly similar results in terms of accuracy. If the emphasis is on ease of calculation or low data requirements the Broad Age SMR Scaling and Broad Age Rate Ratio Scaling methods are good options. They provide a good return for the amount of input data and effort required. TOPALS is also worth considering. If smooth mortality age profiles and a minimal risk of implausible death rates are most important then the Brass Relational and Mortality Surface models work well, though they are more complex to calculate.

Like all forecast evaluation studies, there are of course several limitations which should be acknowledged. The forecast error evaluation extended over a 10-year forecast horizon only due to the limited availability of data on a consistent set of regional boundaries. It was undertaken for SA4 regions of Australia only, and using death rates for period-cohorts with 5-year age widths. All regional to national mortality ratios and parameters were held constant from the base period, implying no changes to relative interregional mortality variations. In reality, there was a modest degree of change to these regional/national ratios over time. For example, regional to national life expectancy at birth ratios in 2001–2006 and 2011–2016 had correlation coefficients of 0.93 for females and 0.95 for males. The selected methods were only assessed against six criteria. Certainly other criteria could have been included, such as those relating to consistency (or coherence) between subnational and national forecasts, and theoretical rigour.

There were also some approximations in the input data, specifically the use of preliminary (rather than finalised) 2016 Estimated Resident Populations in the calculation of death rates due to the finalised data being unavailable at the time of writing. And with all the regional mortality forecasting methods being linked to a national forecast, the results are obviously affected by the accuracy of the national mortality forecast. For this study the national mortality forecast was reasonably accurate. It achieved an Absolute Total Error (overall mortality) of 0.001 in 2006–2011 and 0.006 in 2011–2016; and a Total Absolute Error (mortality age profile) of 0.016 in 2006–2011 and 0.019 in 2011–2016.

Can the findings summarised in Table 6 be generalised to other countries and other types of subnational region? The answer is probably ‘to some extent’. With areas containing populations within the range of SA4 regions (100,000–500,000 people) the results may be broadly similar in other countries, though the extent of interregional variation in mortality will have some impact. For example, a small interregional range in mortality would probably result in the SMR Scaling method generating lower forecast errors overall, with all age-specific death rates lying within the plausible range. A similar assessment of the methods’ performance in other countries would be sensible. Results for smaller geographical regions may produce a wider range of results, especially for local areas where populations and numbers of deaths are smaller. This would usefully be the subject of further research. It would also be beneficial to compare the results of the simpler regional mortality forecasting methods considered here with those of the more complex multi-population forecasting models developed in recent years. More generally, greater research attention on subnational mortality forecasting methods, especially those which meet the needs of practitioners, would be of great benefit.

**Table 6:** Summary evaluation of the mortality forecasting methods applied to Australian regions

Method	Regional input data	Ease of calculation	Ease of assumption setting	Plausibility	Smoothness	Accuracy
National Death Rates	None	Easy	Difficult	High	Smooth	Poor
SMR Scaling	41*r*	Easy	Moderate	Potential problems	Smooth	Poor
Broad Age SMR Scaling	46*r*	Easy	Moderate	Problems unlikely	Smooth	Reasonable
Mortality Surface	2*r*	Complex	Moderate	High	Smoothest	Reasonable
Brass Relational	80*r*	Complex	Moderate	High	Smoothest	Reasonable
Rate Ratio Scaling	80*r*	Easy	Difficult	Potential zero rates	Less smooth	Reasonable
Broad Age Rate Ratio Scaling	12*r*	Easy	Difficult	Problems unlikely	Smooth	Reasonable
TOPALS	80*r*	Moderate	Difficult	Problems unlikely	Smooth	Reasonable

Note: *r* refers to the number of regions

## Abbreviations

ABS: Australian Bureau of Statistics
ATE: Absolute Total Error
ERP: Estimated Resident Population
SMR: Standardised Mortality Ratio
TAE: Total Absolute Error
TOPALS: TOol for Projecting Age-specific rates using Linear Splines

## Footnotes

1 An Excel workbook illustrating the use of all methods (except National Death Rates) is available from the author.

2 Full disclosure: This is the method implemented by the author in several of his population projection programmes (e.g. Wilson 2014, 2015, 2017)
